# Reducing antibiotic use for uncomplicated urinary tract infection in general practice by treatment with uva-ursi (REGATTA) – a double-blind, randomized, controlled comparative effectiveness trial

**DOI:** 10.1186/s12906-018-2266-x

**Published:** 2018-07-03

**Authors:** Kambiz Afshar, Nina Fleischmann, Guido Schmiemann, Jutta Bleidorn, Eva Hummers-Pradier, Tim Friede, Karl Wegscheider, Michael Moore, Ildikó Gágyor

**Affiliations:** 1 Institute for General Practice, Hannover Medical School, Carl-Neuberg-Str. 1, 30625 Hannover, Germany; 2 Department of General Practice, University Medical Center Göttingen, Humboldtallee 38, 37073 Göttingen, Germany; 3 Department for Health Services Research, Institute for Public Health and Nursing Research, University of Bremen, Bremen, Germany; 4 Department of Medical Statistics, University Medical Center Göttingen, Humboldtallee 32, 37073 Göttingen, Germany; 5 Department of Medical Biometry and Epidemiology, University Medical Center Hamburg-Eppendorf, Martinistr, 52, 20246 Hamburg, Germany; 6 Primary Care and Population Science, University of Southampton Faculty of Medicine, Aldermoor Health Centre, Southampton, SO16 5ST UK; 7 Department of General Practice, Universitätsklinikum Wurzburg, Josef-Schneider-Str. 2/D7, 97080 Würzburg, Germany

**Keywords:** Comparative effectiveness design, *Arctostaphylos uva-ursi*, Antibiotic prescription, General practice, Herbal remedy

## Abstract

**Background:**

Uncomplicated urinary tract infections (UTI) are common in general practice and usually treated with antibiotics. This contributes to increasing resistance rates of uropathogenic bacteria. A previous trial showed a reduction of antibiotic use in women with UTI by initial symptomatic treatment with ibuprofen. However, this treatment strategy is not suitable for all women equally. *Arctostaphylos uva-ursi* (UU, bearberry extract arbutin) is a potential alternative treatment. This study aims at investigating whether an initial treatment with UU in women with UTI can reduce antibiotic use without significantly increasing the symptom burden or rate of complications.

**Methods:**

This is a double-blind, randomized, and controlled comparative effectiveness trial. Women between 18 and 75 years with suspected UTI and at least two of the symptoms dysuria, urgency, frequency or lower abdominal pain will be assessed for eligibility in general practice and enrolled into the trial. Participants will receive either a defined daily dose of 3 × 2 arbutin 105 mg for 5 days (intervention) or fosfomycin 3 g once (control). Antibiotic therapy will be provided in the intervention group only if needed, i.e. for women with worsening or persistent symptoms. Two co-primary outcomes are the number of all antibiotic courses regardless of the medical indication from day 0–28, and the symptom burden, defined as a weighted sum of the daily total symptom scores from day 0–7. The trial result is considered positive if superiority of initial treatment with UU is demonstrated with reference to the co-primary outcome number of antibiotic courses and non-inferiority of initial treatment with UU with reference to the co-primary outcome symptom burden.

**Discussion:**

The trial’s aim is to investigate whether initial treatment with UU is a safe and effective alternative treatment strategy in women with UTI. In that case, the results might change the existing treatment strategy in general practice by promoting delayed prescription of antibiotics and a reduction of antibiotic use in primary care.

**Trial registration:**

EudraCT: 2016–000477-21. Clinical trials.gov: NCT03151603 (registered: 10 May 2017).

## Background

Acute urinary tract infections (UTI) represent a common condition in general practice and are usually treated with antibiotics. Though known to be self-limiting in many cases [1–5], UTI account for a significant number of antibiotic prescriptions [6]. This contributes to increasing resistance rates in UTI uropathogenic bacteria and is being discussed critically [7].

Antibiotic prescriptions can be problematic with regard to resistance rates, side effects and costs [8]. Furthermore, several studies show that women with uncomplicated UTI are often willing to delay or even decline antibiotic treatment because they are aware of possible adverse events [5, 9]. Considering these factors, there is a need for evidence of alternative treatment strategies in women with uncomplicated UTI.

In a recent trial, the strategy of initial symptomatic treatment with ibuprofen was proven to be effective in women with uncomplicated UTI and mild to moderate symptom burden [10]. Currently, another randomized controlled trial is being conducted by Vik and colleagues comparing ibuprofen versus mecillinam for uncomplicated cystitis [11]. Since ibuprofen is not suitable for all women equally, other treatment strategies should be explored alternatively. *Arctostaphylos uva-ursi* (UU, bearberry extract arbutin) has traditionally been used to treat UTI symptoms. Former studies have shown antiseptic and antimicrobial properties of UU which are attributed to hydroquinones and tannins [12]. UU is concentrated in the urine and has shown efficacy against bacteria causing UTI [13]. It is safe, only mild adverse events (AE) have been described previously (i.e. gastrointestinal complaints) and detailed investigation did not reveal any toxicity related to the ingestion of UU [14, 15]. In over the counter use, patients should be advised to respect recommended dosage and duration [16]. Being a herbal preparation, a generally high acceptance of UU by patients may be presumed [17]. Limited clinical data from small studies suggest that UU is effective in preventing UTI even in high risk patients [15, 18]. However, its clinical effectiveness in treating acute uncomplicated UTI and its potential to reduce antibiotic use has not yet been subject of a fully powered randomized controlled trial.

The main research questions of this study are:

1. Does initial treatment with UU in women with uncomplicated UTI (starting treatment with UU and prescribing antibiotics only if symptoms persist) reduce the number of antibiotic courses without significantly increasing symptom burden?
2. Is the suggested strategy safe with regard to complications and recurrences?

## Methods

The study protocol of REGATTA is based on the previous trial “Ibuprofen versus fosfomycin for uncomplicated urinary tract infection in women: randomised controlled trial” (ICUTI) [10].

### Study design

REGATTA is a double-blind, randomized, controlled comparative effectiveness trial with active control and parallel groups comparing initial herbal treatment of uncomplicated UTI with immediate antibiotic therapy.

### Trial objectives

#### Co-primary endpoints

Two co-primary endpoints are: 1) number of antibiotic courses day 0–28 and 2) symptom burden (AUC) day 0–7, defined as a weighted sum of the daily total symptom scores, measured as the area under the curve (AUC) of the total symptom score. The trial result is considered positive if superiority of initial treatment with UU is demonstrated with reference to the co-primary outcome number of antibiotic courses and non-inferiority of initial treatment with UU with reference to the co-primary outcome symptom burden. We assume non-inferiority if the symptom burden under UU is increased by less than 25% in comparison to the active control.

#### Key secondary endpoints

With reference to effectiveness: number of early relapses defined as recurrent symptoms until day 14 after initial symptom resolution, number of patients with recurrent UTI day 15–28, defined as recurrent UTI symptoms after initial symptom resolution, number of patients with symptom resolution on day 4 and 7, mean daily symptom sum scores day 0–7, symptom burden (AUC) for individual symptoms (dysuria, urgency, frequency, lower abdominal pain) day 0–7, symptom burden (AUC) day 0–7 of patients with positive and patients with negative urine culture, activity impairment by UTI symptoms days 0–7 (AUC), use of painkillers (defined daily dose, DDD) day 0–7, number of patients taking painkillers, antibiotic use (DDD) day 0–28, number of UTI related visits day 0–28, number of days of UTI related sick leave day 0–28.

With reference to safety: number of patients with temperature > 38 °C day 0–7, number of patients with worsening symptoms, number of patients with prolonged symptoms (> 7 days after inclusion), episodes of pyelonephritis day 0–28 according to general practitioner’s (GP) diagnosis, number of AE and SAE by system organ class day 0–28, proportion of patients with at least 1 AE / 2 AE. All patients will be followed up until symptom resolution. Symptom resolution is defined as max. One score point on each symptom scale.

### Sample size

Demonstrating non-inferiority regarding co-primary endpoint symptom burden drives the sample size. Based on data of the ICUTI trial [10], it was assumed that the coefficient of variation of symptom burden will be 70%. With this coefficient of variation the sample size required to demonstrate non-inferiority as defined above at a one-sided significance level of 2.5% with a power of 90% will be 170 patients per group, i.e. Three hundred forty patients in total give no difference in symptom burden between the groups. A Wilcoxon rank sum test comparing the two treatment groups with regard to the co-primary endpoint number of antibiotic courses has a power of at least 90% given the sample size of 170 patients per group and a probability of at least 61% that a patient in the intervention group takes fewer antibiotic courses than a patient in the control group. This is a conservative assumption as this probability was estimated to be in excess of 80% from data in the ICUTI study [10]. Adjusting for a drop-out rate of 20%, 430 patients have to be randomized. The sample sizes were calculated using nQuery Advisor® Version 7.0.

### Trial population

#### Setting and recruitment

General practices in Germany (Lower Saxony, Hesse, North-Rhine Westphalia, Thuringia and Bremen) will participate in the trial and recruit 430 patients during a 16 months recruitment period. The academic study centers in Göttingen and Hannover will provide structured practice support to optimize patient recruitment with newsletters, telephone calls and incentives.

On site monitoring visits are planned at the beginning and during the study for source data verification and to ensure correct procedures and documentation.

#### Inclusion and exclusion criteria

Women between 18 and 75 years with suspected UTI presenting at general practice with at least two of the following symptoms: dysuria, urgency, frequency and lower abdominal pain will be asked for participation, assessed for eligibility and included after written informed consent.

Key exclusion criteria comprise any signs of complicated UTI (i.e. temperature > 38 °C, loin tenderness), any conditions that may lead to complicated infections (i.e. renal diseases, patients with urinary catheter), pregnancy or breastfeeding, current self-medication with UU preparations, antibiotic use in the last 7 days, previous UTI in the past 2 weeks, history of pyelonephritis, contraindications for trial drugs, severe diseases (i.e. serious infection, multiple sclerosis), inability to understand trial information, and current participation in another clinical trial.

#### Trial drug and interventions

UU is a dry extract (dry extract ratio 2.5–4.5:1), extraction solvent water, containing 20–28% of hydroquinone derivatives calculated as anhydrous arbutin (spectrophotometry). The dosage for UU will be 3 × 2 105 mg arbutin for 5–6 days until all tablets have been completed. This will give a daily total of 630 mg arbutin, which is below the maximum dose of 840 mg as recommended by the European Medicines Agency (EMA). Haupt Pharma Wülfing GmbH, Member of the Aenova Group, is charged with the production of the UU tablets (Arctuvan®) and with the repacking of the tablets in new blisters. Six tablets will be packed in a blister and 5 blisters will be packed into a box. The blisters will be labeled by the Hospital Pharmacy of the University Hospital of Schleswig-Holstein (UKSH) who will prepare the drug as a clinical trial product. The shelf life is 3 years.

After written informed consent, patients receive either antibiotic therapy with fosfomycin 1 × 3 g or initial treatment with UU tablets (3 × 2 105 mg arbutin) for 5 days. In case of persistent or worsening symptoms, specific antibiotic treatment in line with the results of the urine culture can be initiated at the discretion of the GP. Since fosfomycin is only orally available as granules, a double dummy design is planned. The intervention group takes placebo granule sachets once additional to UU tablets (3 × 2 105 mg arbutin) whereas the control group takes placebo tablets 3 × 2 for 5 days additional to fosfomycin. Patients will be instructed to take preexisting co-medication as usual. Co-medication as well as any analgesics or other additional drugs will be documented in the electronic case report form (eCRF).

Randomization will be performed on patient level. Drug units will be labelled with code numbers from a computer generated random list. At inclusion, patients receive a drug unit, and the code number from the drug unit will be assigned to the patient.

### Clinical trial procedures

At inclusion (day 0), patients complete a symptom questionnaire after informed consent. Further, patients provide a urine specimen for dipstick, culture and pregnancy test, and body temperature will be taken in the ear or orally. GPs hand over the drug unit and recommend patients to visit again if symptoms persist or worsen or if fever occurs. If patients return therapy will be changed at the discretion of the GP and according to the German UTI guideline [19], results of the microbiological tests will be available after 4–5 days. Native urine samples will be stored in the refrigerator max. 24 h until collection to the laboratory. All urine cultures will be performed in one central laboratory.

### Data collection and management

Participant timeline in REGATTA with information on schedule for enrolment, interventions, assessments, and visits is provided in Table 1.

**Table 1:** Participant timeline in REGATTA

Activity	Patient Contact	Baseline	Follow-up							
Day		0	1	2	3	4	5	6	7	28
Visit Number		I								II
Screening		X								
Informed Consent		X								
Inclusion / Exclusion criteria		X								
Medical History, Examination		X								
Urine Tests^a^		X								
Pregnancy Test		X								
Drug Intake^b^		X	X	X	X	X	X			
Symptom Assessment and Activity Impairment (Diary)		X	X	X	X	X	X	X	X	
Additional Antibiotic Courses			X	X	X	X	X	X	X	X
Painkiller Intake			X	X	X	X	X	X	X	X
Adverse Events and Complications			X	X	X	X	X	X	X	X
Recurrent UTI (Questionnaire)										X
Return of Drug Packages										X
Phone Calls to Remind Patients (Diary, Drug Return, Questionnaire)			X						X	X

^a^Midstream urine for on-site dipstick test (leukocytes, nitrite, red blood cells) and for urine culture (pathogens and resistances)

^b^Drug intake until all 30 tablets are finished (= 3 × 2 tablets uva-ursi daily for 5 days)

#### Inclusion: Symptom questionnaire

Patients will complete a symptom questionnaire which has been used in previous UTI studies but has not been validated yet [4, 10]. Duration and severity of symptoms and activity impairment will be documented. Symptom evaluation will cover the symptoms dysuria, urgency, frequency and lower abdominal pain, each scored from 0 (none) to 4 (very strong). UTI-related activity impairment covers impairment by each single symptom (see above), scored as well from 0 (none) to 4 (very strong) [20]. Data will be transferred to the web-based eCRF by practice staff.

#### Follow-up

Patients will be asked to record daily symptom severity and activity impairment in the patient diary for at least 7 days. Additionally, pain killer intake and any antibiotic treatment between day 0–7 will be documented. The diaries will be sent back by the patients to the general practice. There will be a follow-up documentation on day 28, where patients will complete the follow-up survey including antibiotic intake, relapses and recurrent UTI, AEs/SAEs, UTI-related consultations and days of sick leave. Study nurses will transfer these data to the web-based eCRF.

### Statistical analysis and reporting

The primary analysis will be based on the results of two statistical tests corresponding to two co-primary endpoints. The first test will be on the following two hypotheses H0 (H1): The rate of antibiotic courses per patient within the interval 0–28 days in the UU group is greater or equal (lower) than the rate in the fosfomycin group. The second test will be on the following two hypotheses: H0 (H1): The symptom burden within the interval 0–7 days in the UU group is greater or equal (lower) than 125% of the corresponding symptom burden in the fosfomycin group. Since both criteria have to be fulfilled for the study to be positive, both hypotheses are tested at a one-sided level of 2.5% and the overall type I error rate will still be controlled at the one-sided level of 2.5% (intersection-union method).

The number of antibiotic courses within the interval 0–28 days will be compared between treatment groups using a Wilcoxon rank sum test. The treatment effect will be reported in terms of the so-called relative effect (or also probabilistic index) with 95% confidence interval [21]. As supporting analyses, the number of antibiotic courses will be modelled using suitable parametric models such as negative binomial regressions with treatment group and study center as factors and baseline symptom score as covariate.

An analysis of covariance (ANCOVA) of log symptom burden will be performed with the treatment group as factor and the day-0 (inclusion) log sum of symptom scores as covariate. From this model, a two-sided 95% confidence interval for the ratio of the expected total symptom burden of conditional antibiotic use vs. immediate use will be derived and compared to the non-inferiority margin of 125%.

Secondary endpoints will be explored using regression models appropriate for type of scale, without adjustment for multiplicity.

### Patient safety

At inclusion, patients will be advised to consult their GPs at any time in case of ongoing or worsening symptoms. In this case, specific antibiotic therapy can be provided as soon as the resistogram of the urine culture taken at inclusion is available. Adverse events (AE) leading to consultation will be documented in the eCRF by the GP. Serious AE (SAE) will have to be reported by fax within 24 h after becoming aware of it. An independent Data and Safety Monitoring Board (DSMB) will be established to assess safety risks based on the safety related data regularly. In case of cumulative occurrence of SAE or pyelonephritis, the DSMB will decide whether to continue or discontinue the trial.

### Ethical considerations

Ethical approval has been obtained by the Independent Ethics Committee of the University Medical Center Göttingen (No. 16/11/16). The study will be conducted according to the principles of Good Clinical Practice (GCP). The patient information sheet has been developed according to current ethic committee’s standards. At inclusion, GPs ensure complete orally information about risks, benefits, and study procedures, and take patients’ informed consent. Patients declare their agreement to disclosure of pseudonymized data based on current data protection regulations. All patient related data will be treated confidentially. Patients can withdraw their consent for the trial at any time.

This trial is ethically justifiable since harm is unlikely due to the good prognosis of the condition and the use of known and authorized medicines for a period of only 5 days. Additionally, patients will be informed that if their symptoms persist or reoccur they can return to the practice at any time and that, if needed, specific antibiotic treatment can be initiated.

The benefit for individuals comprises saving patients from unnecessary antibiotic treatment and possible antibiotic side effects. In general, the reduction of unnecessary antibiotic prescriptions helps to decrease resistance development.

### Patient involvement

We use different approaches to include the patients’ perspectives into the design and conduction of this trial.

#### Patient board

A patient board involving 10 patients with previous UTI will accompany all study procedures, some board members have already participated in the previous trial on UTI [10]. The patient board meets on a regular basis and is involved in the discussion on study documents and material. They will also contribute their perspectives to assist the recruitment of patients, discuss outcomes and support the implementation of results.

#### Involvement of patient representatives

The patient representative involved in the update of the German guideline on UTI [19], approved the REGATTA trial, its design, endpoints and patient-related aspects. No further changes in the protocol were requested.

#### Previous experiences

Patients of the previous ICUTI trial have been interviewed after study participation [22]. They pointed out the importance of feeling “safe” in the trial with regard to reliable symptom relief. These results were considered in the planning of REGATTA. Consequently, we do not have a placebo arm but remain with a two-active-treatments design. Furthermore, as patients appreciated in ICUTI, GPs will recommend consultation in cases of persistent symptoms.

The concept to delay antibiotic therapy is supported by different international studies [5, 23]. In a Dutch cohort study for instance [5] one third of women with suspected UTI were willing to delay antibiotic therapy – although in this study no alternative therapy besides watchful waiting was provided.

### Registration

This study is registered at clinical trials.gov (NCT03151603) with the acronym REGATTA.

## Discussion

This study will show if the use of antibiotics for uncomplicated UTI can be reduced by this alternative treatment. We want to investigate whether the initial treatment with UU is a safe and effective alternative treatment strategy in women with uncomplicated UTI. By choosing a comparative effectiveness design, we will be able to prove the effectiveness of two therapeutic strategies and not only the drug efficacy.

REGATTA is designed to create evidence for an alternative treatment option in UTI and to provide information about the clinical effectiveness and potential to reduce antibiotic use. The study results can indicate an alternative treatment for women who are willing to avoid antibiotic treatment, and may possibly change the management of UTI by this approach that fits perfectly in the daily routines of primary care. In contrast to many UTI trials focusing on patients with microbiologically proven UTI, this study, similarly to the previous study ICUTI, follows a more pragmatic approach by including patients presenting with typical symptoms. The study sample of patients with uncomplicated UTI who are otherwise healthy represent a typical practice population. Thus, external validity is high and results can easily be transferred to routine in general practice.

To assess bacterial count and species, urine cultures will be provided at inclusion for trial reasons only. The results will allow a distinction between patients with and without bacterial infections in the analysis and provide data on resistance in case a secondary antibiotic treatment is needed. Again, this represents a pragmatic approach since treatment decisions for uncomplicated UTI are usually made without microbiological specification in general practice.

### Trial objectives

In this study, we aim to investigate benefits and potential risks of two different treatment strategies in UTI. The benefit will consist of a reduced number of antibiotic prescriptions which implies a decrease of resistance rates, side effects and costs. Simultaneously, a potential risk of higher symptom burden will be considered very carefully. Therefore, two co-primary endpoints were chosen to reflect both aspects. Additionally, safety criteria will be assessed by several safety endpoints such as patients with poor outcomes, UTI recurrences and complications, pyelonephritis and number of AE and SAE up to day 28.

### Trial drug

UU has antiseptic and antimicrobial properties which are attributed to hydroquinones and tannins [12]. UU is concentrated in the urine and has shown efficacy against bacteria causing UTI [13]. Since there is only few data about the safety and efficacy of UU in uncomplicated UTI there is a need for further investigation. A similar trial investigates whether UU compared to placebo and the advice to take ibuprofen compared to no advice provides relief from urinary symptoms in women with uncomplicated UTI. Results of this study and of REGATTA will provide evidence of strategies to reduce antibiotic use [24].

The control group in REGATTA will be treated with fosfomycin as recommended by the German UTI guideline as one of the first line treatment options in uncomplicated UTI [19]. The resistance rates of fosfomycin with respect to *E. coli* are low [5] and the treatment of symptoms is effective. According to product information and guideline recommendations a single dose treatment is sufficient in UTI [25–27].

As UTI may resolve spontaneously, a third group with observation only could generate further data on the effects of a watch and wait treatment strategy. However, this might also lead to a less participation rate. Patients in this study will receive an active substance irrespective of which group they are randomized to.

### Trial procedures

Uncomplicated UTIs have a good prognosis and usually do not require follow-up consultations. To follow this pragmatic approach and to influence the course of the symptoms as little as possible, only one final visit at day 28 without a GP contact is planned in this study. Return visits are possible at any time if symptoms persist or reoccur.

Study-related changes of usual GP procedures are minimized in order to optimize external validity. Nevertheless, GPs can decide whether further diagnostic procedures or an alteration of the initial treatment strategy is necessary in patients with i.e. persistent or worsening symptoms. In this study we tried to keep GPs’ and patients’ effort as simple and reasonable as possible. Results of a qualitative study of physicians’ experiences with a clinical trial confirm that trial procedures should be as simple as possible to successfully implement a clinical trial in family medicine [28]. Therefore, additional procedures (i.e. measurement of the fluid intake or ultrasound for residual urine or renal calculi) were not assessed.

### Patient safety

No trial-related invasive procedures are planned. A urine culture will be performed at inclusion so that specific antibiotic therapy can be initiated if necessary in case of persistent or recurrent symptoms.

We estimate that adverse drug events will occur less frequently with UU than with antibiotic therapy and we do not expect complicated disease courses, since UTI is a benign condition. Besides, in both groups the treatment courses are very short.

Although only few data exist, the risk of pyelonephritis after non-antibiotic treatment of UTI is mentioned frequently [3, 29]. In ICUTI comparing ibuprofen versus fosfomycin for uncomplicated UTI, only 5 of 241 patients treated with ibuprofen were suspected to have a pyelonephritis [10]. We expect that some patients will require antibiotic treatment after failure of non-antibiotic treatment, but this will be predominated by the benefits of patients with symptom resolution without antibiotics. Eventually, the rate of relapses might increase – this will be assessed within the trial. The incidence of pyelonephritis/ febrile UTI and poor outcomes in both groups will be monitored. In a follow-up study, we will assess the number of patients with recurrent UTI or pyelonephritis after 3 months.

## Conclusions

A confirmation of the trial hypothesis could lead to a revision of the recommended treatment for uncomplicated UTI. Using UU as a first line treatment option may be proven as effective in resolution of UTI symptoms and reduction of antibiotic use. It may also provide favorable effect on resistance rates.

## Abbreviations

AE: Adverse event
AUC: Area under curve
DDD: Defined daily dose
eCRF: Electronic case report form
GP: General practitioner
ITT: Intention to treat
PP: Per protocol
SAE: Serious adverse event
UTI: Urinary tract infection
UU: Uva-ursi
